# The gonadal transcriptome of the unisexual Amazon molly *Poecilia formosa* in comparison to its sexual ancestors, *Poecilia mexicana* and *Poecilia latipinna*

**DOI:** 10.1186/s12864-017-4382-2

**Published:** 2018-01-03

**Authors:** Ina Maria Schedina, Detlef Groth, Ingo Schlupp, Ralph Tiedemann

**Affiliations:** 1 Unit of Evolutionary Biology/Systematic Zoology, Institute of Biochemistry and Biology, University of Potsdam, Karl-Liebknecht-Straße 24-25, Haus 26, 14476 Potsdam, Germany; 2 Department of Bioinformatics, Institute of Biochemistry and Biology, University of Potsdam, Karl-Liebknecht-Straße 24-25, Haus 14, 14476 Potsdam, Germany; 3 Department of Biology, University of Oklahoma, 730 Van Vleet Oval, Norman, OK 73019 USA

**Keywords:** Differential gene expression, Gynogenesis, Hybrid speciation, Meiosis, *Poecilia formosa*, *Poecilia latipinna*, *Poecilia mexicana*

## Abstract

**Background:**

The unisexual Amazon molly (*Poecilia formosa*) originated from a hybridization between two sexual species, the sailfin molly (*Poecilia latipinna*) and the Atlantic molly (*Poecilia mexicana*). The Amazon molly reproduces clonally via sperm-dependent parthenogenesis (gynogenesis), in which the sperm of closely related species triggers embryogenesis of the apomictic oocytes, but typically does not contribute genetic material to the next generation. We compare for the first time the gonadal transcriptome of the Amazon molly to those of both ancestral species, *P. mexicana* and *P. latipinna*.

**Results:**

We sequenced the gonadal transcriptomes of the *P. formosa* and its parental species *P. mexicana* and *P. latipinna* using Illumina RNA-sequencing techniques (paired-end, 100 bp). De novo assembly of about 50 million raw read pairs for each species was performed using Trinity, yielding 106,922 transcripts for *P. formosa*, 115,175 for *P. latipinna*, and 133,025 for *P. mexicana* after eliminating contaminations. On the basis of sequence similarity comparisons to other teleost species and the UniProt databases, functional annotation, and differential expression analysis, we demonstrate the similarity of the transcriptomes among the three species. More than 40% of the transcripts for each species were functionally annotated and about 70% were assigned to orthologous genes of a closely related species. Differential expression analysis between the sexual and unisexual species uncovered 2035 up-regulated and 564 down-regulated genes in *P. formosa*. This was exemplary validated for six genes by qRT-PCR.

**Conclusions:**

We identified more than 130 genes related to meiosis and reproduction within the apomictically reproducing *P*. *formosa*. Overall expression of these genes seems to be down-regulated in the *P*. *formosa* transcriptome compared to both ancestral species (i.e., 106 genes down-regulated, 29 up-regulated). A further 35 meiosis and reproduction related genes were not found in the *P*. *formosa* transcriptome, but were only expressed in the sexual species. Our data support the hypothesis of general down-regulation of meiosis-related genes in the apomictic Amazon molly. Furthermore, the obtained dataset and identified gene catalog will serve as a resource for future research on the molecular mechanisms behind the reproductive mode of this unisexual species.

**Electronic supplementary material:**

The online version of this article (10.1186/s12864-017-4382-2) contains supplementary material, which is available to authorized users.

## Background

Sexual reproduction is the most common form of reproduction in the animal kingdom, and only 0.1% of all animal species reproduce asexually [1]. Compared to asexual reproduction, sexual reproduction enables genetic recombination, but seems otherwise to be less efficient and exhibits profound costs, like the two-fold costs of males [2]. The evolution, persistence and underlying molecular mechanisms of both sexual and asexual reproduction are therefore central topics of evolutionary biology [3, 4]. In sexually reproducing eukaryotes, meiosis, the reduction division of diploid germ cells to generate haploid gametes such as sperm, eggs, and pollen, is an essential process. After fertilization, zygotes are created by incorporating the genetic material of both sexes, restoring the original ploidy level. This is in contrast to some asexual species, including the Amazon molly (*Poecilia formosa*) where no meiotic cell cycle takes place and the gametes are produced via mitosis [5]. There are several variants and types of asexual reproduction, but we will focus here on the prevalent type in vertebrates, which is parthenogenesis. In many species with parthenogenesis, meiosis is lacking (apomixis) and oocytes do not undergo a reduction division leading to diploid eggs [6]. Consequently, offspring are genetically identical to the mother. In vertebrates, this phenomenon is found in fishes, amphibians, and reptiles and only known for species of hybrid origin [7], shedding light on the role of hybridization in functional aspects of biology, and in particular in hybrid speciation. These unisexual vertebrates are used as model organisms to understand the origin and maintenance of sexual reproduction and meiosis. However, the underlying mechanisms driving asexuality, as well as the mechanisms of the transition from sexuality to asexuality, are still unclear.

Meiosis and sexual reproduction seem to have arisen very early in eukaryotic evolution and therefore vertebrate asexual lineages originated from sexual relatives [8]. Schurko and Logsdon Jr. [9] propose that the presence of a set of multiple genes required specifically for meiosis is indicative of the capability of an organism to undergo meiosis and should imply sexual reproduction. In the genome of an apomictic species, these genes should be obsolete and undergo genomic decay to the point where they are dysfunctional. Alternatively, they may evolve other functions. Meiosis genes were detected even in the putative ancient asexual protists *Giardia intestinalis* [8] and *Trichomonas vaginalis* [10]. Recently evolved asexual species, such as apomictic hybrid species provide an excellent model to investigate the evolution of meiosis-related genes under presumably relaxed selective constraints and can help to understand the transition from sexuality to asexuality. The unisexual hybrid species *P. formosa* and its bisexual, parental species are a particularly suitable model to investigate differences between sexually and asexually reproducing species and to determine relevant genes for the underlying processes.

*P. formosa* derived its common name ‘Amazon molly’ from a mythological Greek tribe of warrior women, the Amazons. It is an all-female species [11] with a natural distribution in the coastal areas along Northeastern Mexico and Southern Texas [12]. It reproduces clonally by sperm-dependent parthenogenesis, i.e., gynogenesis [13, 14]. Although this is a mode of asexual reproduction, gynogenesis does involve the mating of a female with a male from a different species (pseudo-fertilization) [15]. *P. formosa* evolved by hybridization of two sexually reproducing species, the Atlantic molly *Poecilia mexicana* (maternal) and the sailfin molly *Poecilia latipinna* (paternal) [16–18], and originated around 280, 000 years ago [19]. Both ancestral species [11] and the very closely related Tamesí molly (*Poecilia latipunctata*) [20] can act as sperm donors for *P. formosa* to initiate embryogenesis of the diploid apomictically produced oocytes [21, 22]. *P. formosa* progeny are identical copies (clones) of the mother, since the genetic material of the sperm donors does not contribute to the offspring except in very rarely occurring events of paternal introgression [23, 24], when parts of or the complete genetic material of the sperm introgresses and is passed on to subsequent generations, leading to polyploid or microchromosome-bearing lineages [25]. In the family Poeciliidae, like in other live-bearing fishes, insemination takes place by introducing the sperm via a copulatory organ, the modified primary anal fin (gonopodium), to the reproductive tract of the females [26]. Therefore, *P. formosa* must occur in sympatry with at least one of the species acting as sperm donors to be able to mate and subsequently reproduce [7]. This behavior has been described as sexual parasitism, given that the males gain no apparent benefits from mating with the heterospecific *P. formosa* [27], except under mate copying scenarios described by Schlupp et al. [28] and Heubel et al. [29].

In this study, we focus on the detection of genes that encode components specific for reproduction and meiosis. The presence or absence of functional (i.e., expressed) copies of these genes is evaluated by comparative transcriptome analyses of the unisexual Amazon molly *P. formosa* and its parental bisexual species. Such analysis can help to resolve the underlying molecular processes between the two reproduction modes and their evolution. Transcriptomics are a common tool for identifying genes of interest (candidate genes) for diverse research topics [30] and are particularly suitable to discover unique and shared genes/gene expression among closely related species [31, 32]. Here, we describe and characterize the transcriptome of a hybrid vertebrate, *P. formosa,* in comparison to both ancestral sexual species, *P. latipinna* and *P. mexicana*, generated by high-throughput sequencing of RNA from the gonads. The identification of more than 100 expressed genes related to reproduction, especially the meiotic cell cycle, in an apomictic species is remarkable and will provide a valuable genomic resource for future studies.

## Methods

### Sample preparation and next generation sequencing

To construct the transcriptomes of all three species (*P. formosa*, *P. mexicana,* and *P. latipinna*) the gonadal RNA of three females per species was sequenced with next-generation sequencing methods. These fish were taken from strains kept and bred at the University of Potsdam (Germany). The founder individuals of *P*. *formosa* (strain For III/9) were collected at Río Purificación (Barretal, Tamaulipas, Mexico) in 1993, *P*. *latipinna* (strain F.O II/7 1355) at Key Largo (Florida, USA) in 1993, and *P*. *mexicana* (strain Mex IV/5) at Laguna de Champaxan (Altamira, Tamaulipas, Mexico) in 1994. The fish were kept under standard conditions (12:12 h light-dark cycle at 25 °C) at the University of Potsdam in compliance with German animal welfare regulations. Two months before tissue collection (which took place in 2013), sexually mature females of each species were isolated into separate tanks to avoid gene expression shifts due to interactions with males. Before sacrificing the fish on ice, the sex and species affiliation of each individual was verified by examining the anal fin structure and the dorsal fin ray number, respectively. The excised gonads were immediately frozen in liquid nitrogen and stored at −80 °C. For RNA extraction, a combination of Trizol (Life Technologies) and the RNeasy Mini Kit (Qiagen) extraction methods was performed, including a genomic DNA removal protocol. Detailed instructions for the tissue collection and RNA isolation procedure can be found in Zhu et al. [33]. The total yield of RNA was calculated by measuring the concentration and purity using a Spectrophotometer (NanoDrop 1000; ThermoScientific) and the RNA isolates of three individuals per species were pooled for library preparation. A commercial sequencing provider (LGC Genomics GmbH, Berlin) performed transcriptomics library preparation and sequencing (100 bp, paired-end) of all three libraries on one channel of an Illumina HiSeq2000, as well as demultiplexing and adapter clipping (Casava v1.8.2; Illumina Inc.).

### Preprocessing – Quality control, filtering and trimming

The initial processing of the data included quality control, filtering, and trimming of the raw reads. After controlling the quality of the obtained paired-end reads with the FastQC software (v0.11.2) [34], we used Trimmomatic (v0.32) [35] to perform different filtering and trimming steps. First, all reads containing an unknown base character (`N`) were removed. Second, bases which showed a low quality at the start or end of the read were cut off (leading/trailing). Third, the sliding window algorithm scanned the reads with a specific base wide sliding window (4 bp), which cut off when the average quality per base dropped below an average quality threshold (15). After trimming, the potentially present ribosomal RNA (rRNA) fragments were excluded from the dataset with SortMeRNA (v2.0) [36]. This software filters and removes rRNA by comparing the reads with clustered rRNA sequence databases of the small and large subunits of bacteria, eukaryotes, and archaea, compiled with the data of the SILVA project [37].

### De novo assembly and removal of contamination

We initially built the transcriptomes on the basis of two strategies, de novo and genome-guided with the genome of *P. formosa* as reference genome (Ensemble release 2014) [38]. Recently, it has been argued that reference genomes are not always well suited as references for RNA sequencing experiments, unless they have been re-annotated before [39, 40]. Indeed, our assembly statistics and functional annotations for the reference-guided assemblies were not as good as the individual de novo assemblies for the three species. Therefore, we used the de novo assemblies for all subsequent analyses. The assembly of the trimmed and filtered reads was done with the software package Trinity (r20140717 to v2.2.0) [41] with standard parameters. Trinity is a widely used assembler based on the method of *de Bruijn* graphs for the reconstruction of transcriptomes de novo or genome-guided from RNA sequencing data. The Trinity assembler comprises three major consecutive software modules: First, reads were combined into larger contigs (by Inchworm), second, these contigs were clustered into components (by Chrysalis), and finally the most plausible sets of transcripts from these groups were produced (by Butterfly). Downstream analyses, e.g., to calculate quality statistics of the transcriptomes were conducted with the associated software tools of Trinity using Bowtie2 (v2.2.24) [42], SAMtools (v1.3) [43], and RSEM [44]. All sequence comparisons were conducted with the Basic Local Alignment Search Tool (BLAST) (v2.3.0+ and v2.6.0+) [45]. To identify potential contaminants within the assemblies, the transcripts of all the species were compared to protein sequence databases of four different non-target taxa (archaea, bacteria, fungi, and invertebrates) in UniProt (Swiss-Prot/TrEMBL release 2014_10) [46]. Beforehand, each taxonomic database was clustered by removing redundant sequences with 95% identity (CD-Hit v4.6.1) [47]. Transcripts which had a match were compared against a protein database of *Danio rerio* (TrEMBLE release 2014_10) to ensure that only real contaminants were eliminated from further analyses, while transcripts showing a high similarity with a fish sequence database were retained. Also, transcripts missing an open reading frame (ORF) were removed. ORF identification was achieved using the web server of OrfPredictor (v2.3) [48].

### Annotation and comparative analyses

Classification of gene ontology (GO) terms into the categories “biological process”, “cellular components”, and “molecular functions” associated with a given gene product was carried out with the standalone graphical user interface (GUI) version of GOblet (v0.2.1) [49]. Based on sequence similarities and comparisons to well-annotated proteins from UniProt databases, the contigs of all three species were annotated with terms from the Gene Ontology project [50]. Only records with evidence codes assigned by curators of the GO Consortium from the UniProt/Swiss-Prot databases (release 2015_06) of humans, rodents, vertebrates, and mammals were chosen with an E-value cut-off of 1e^−10^, while those inferred solely from electronic annotation (IEA) were not considered. For each assembly, the frequencies of occurrence for the 150 generic GO slim terms (www.geneontology.org/ontology/subsets/) were calculated. The generic GO slim terms developed by the GO Consortium contain those GO terms, which show a high biological relevance and cover most of the genes/proteins annotated for all species in the database. Species-specific over- and under-representation of the GO terms was tested with a Fisher’s exact test (α = 0.05) with false discovery rate (FDR) correction of the *p*-value.

We conducted several sequence comparisons with different protein, genomic, and complementary DNA (cDNA) datasets of teleost fish species (Additional file 1: Table S1; E-value cut-off: 1e^−50^) [51] and the UniProt/Swiss-Prot database (release 2015_03; E-value cut-off: 1e^−20^) using the BLAST algorithm. For the identification of candidate genes relevant to our focus on sexual *vs*. asexual reproduction, the results were scanned for genes known to be involved in meiosis [8–10, 52, 53]. Furthermore, transcripts were translated to amino acid sequences with a minimum length of 70, using the Transdecoder pipeline (v3.0.2; http://transdecoder.github.io), which identifies coding regions and detects the longest ORF for every transcript in combination with homology results from the Swiss-Prot database (E-value cut-off: 1e-5) and Hmmer (v3.1b2) [54], which searches the peptides for protein domains against the pfam database (release 30.0) [55], a collection of protein family alignments. These sets of amino acid sequences were further analyzed by the OrthoFinder pipeline (v1.1.4) [56] to identify orthogroups of the three assemblies, using the *Poecilia reticulata* proteome as reference [57]. Afterward, the orthogroups were annotated with GOblet and analyzed specifically with regard to differences between the unisexual *P. formosa* and the three sexual species (*P. latipinna*, *P. mexicana,* and *P. reticulata*).

### Differential expression

Processed reads of each species were mapped back to the combined transcripts of all three species with Bowtie2 using strict mapping parameters (no-discordant | no-mixed | score-min L,-0.1,-0.1). Then, the transcripts were clustered with Corest (v1.06) [58] and for each gene cluster, the number of mapped reads of each species was compiled. Based on the clustering, differential expression between the unisexual (*P. formosa*) and sexual (*P. mexicana* and *P. latipinna*) species was analyzed for gene clusters with transcripts occurring in all three species using edgeR [59]. Because of the absence of a second (replicate) unisexual species, the dispersion value was set to 0.1 as recommended in the manual and statistical significance of inferred up- and down-regulated genes was not evaluated. Up- and down-regulated genes were annotated with GOblet (v0.2.2). For specific parent GO terms and all their child-terms, all entries were manually scanned to identify further candidate genes to be differentially expressed under unisexual *vs*. sexual reproduction. For six genes related to the androgen receptor pathway, we obtained gene-specific expression data produced by quantitative real-time RT-PCR (abbreviated as qRT-PCR; data from [33]), originating from the same RNA isolates used for our transcriptome analysis. These gene-specific expression levels were used to exemplary validate differential expression of the respective genes derived from the transcriptome data.

## Results

### Next generation sequencing and de novo assembly

The sequencing of the three individuals from each species, pooled into one library on one lane of an Illumina HiSeq2000 (paired-end, 100 bp), yielded 115,183,830 raw reads for the Amazon molly *P. formosa*, 117,678,742 for the sailfin molly *P. latipinna*, and 100,309,634 for the Atlantic molly *P. mexicana* (Table 1). The quality control with FastQC showed that the Phred quality was lower in the first three base pairs of the reads, as well as at the end. After adapter clipping and trimming, the number of read pairs was 56,916,341 for *P. formosa*, 58,302,260 for *P. latipinna,* and 49,722,788 for *P. mexicana*. The reads had an average length of 94 bp for *P. formosa* and 95 bp for *P. latipinna* and *P. mexicana*. The average Phred quality of the reads was about 36 (Table 1). Before the assembly, the read pairs which presented potential rRNA fragments were removed with SortMeRNA (0.49% for *P. formosa* | 0.85% for *P. latipinna |* 0.89% for *P. mexicana)*. The processed reads for all three species can be obtained from the Sequence Read Archive (SRA) at the National Center for Biotechnology Information (NCBI) (BioProject: PRJNA385580 - *P. formosa*: SAMN06894540 | *P. latipinna*: SAMN06894541 | *P. mexicana*: SAMN06894542).

**Table 1:** Transcriptome sequencing results (100 bp, single end)

Species	*Poecilia formosa*	*Poecilia latipinna*	*Poecilia mexicana*
Raw reads	115,183,830	117,678,742	100,309,634
Adapter clipped reads	115,141,762	117,644,190	100,280,696
Adapter clipped read pairs	57,570,881	58,822,095	50,140,348
Total bases	29,987,059,554	30,641,028,948	26,118,870,016
Total read pairs^a^	56,916,341	58,302,260	49,722,788
Average read length^a^	94	95	95
Average Phred quality^a^	35.7	35.8	35.9

^a^: After trimming

The de novo assembly for the three read sets was conducted with the Trinity assembler (Table 2). The average contig length for the 108,690 transcripts for *P. formosa* was 1077 bp, for *P. latpinna* there were 117,211 transcripts with an average length of 1232 bp, and for *P. mexicana* the average length was 1365 bp across 135,217 transcripts (Additional file 2: Figure S1). The weighted median length of the transcripts (N50 value) was 1764 bp for *P. formosa*, 2339 bp for *P. latipinna*, and 2569 bp for *P. mexicana*. By comparisons with the four clustered databases of archaea, bacteria, fungi and invertebrates (clustering reduction: bacteria 81%, archaea 27%, fungi and invertebrates 32%) from UniProt (Swiss-Prot/TrEMBL), we detected 1106 (1.02%) possible contaminants among *P. formosa* transcripts, 1160 (0.99%) in *P. latipinna* and 1209 (0.89%) in *P. mexicana*, mostly belonging to invertebrates. ORFs were missing in 0.61% of the transcripts of *P. formosa*, 0.75% in *P. latipinna*, and 0.73% in *P. mexicana*. In total 1768 (1.63%) contigs for *P. formosa*, 2035 (1.74%) for *P. latipinna,* and 2192 (1.62%) for *P. mexicana* were excluded from the transcriptome datasets used for further analysis, either as contaminants or because of a lacking ORF.

**Table 2:** Statistics for the de novo assembly

Species	*Poecilia formosa*	*Poecilia latipinna*	*Poecilia mexicana*
Transcripts	108,690	117,211	135,217
Components	59,935	73,450	79,522
Total number of base pairs	117,095,092	144,420,105	184,588,701
Average contig length (bp)	1077	1232	1365
Median contig length (bp)	682	625	713
N50 (bp)	1764	2339	2569

### Comparative analysis and identification of candidate genes

**Fig. 1:**
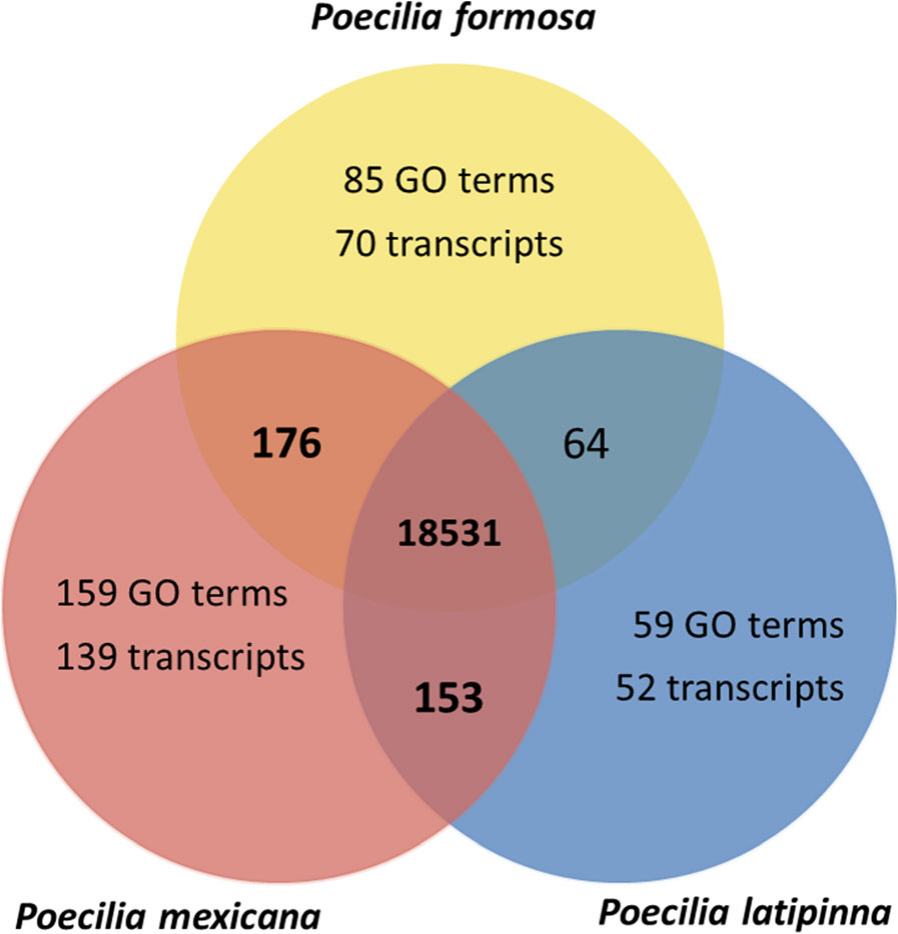
Venn diagram of unique and shared gene ontology (GO) terms for the three species Total numbers of identified GOs unique for each species and shared among species; for GOs unique to a species, the respective number of transcripts are specified

**Fig. 2:**
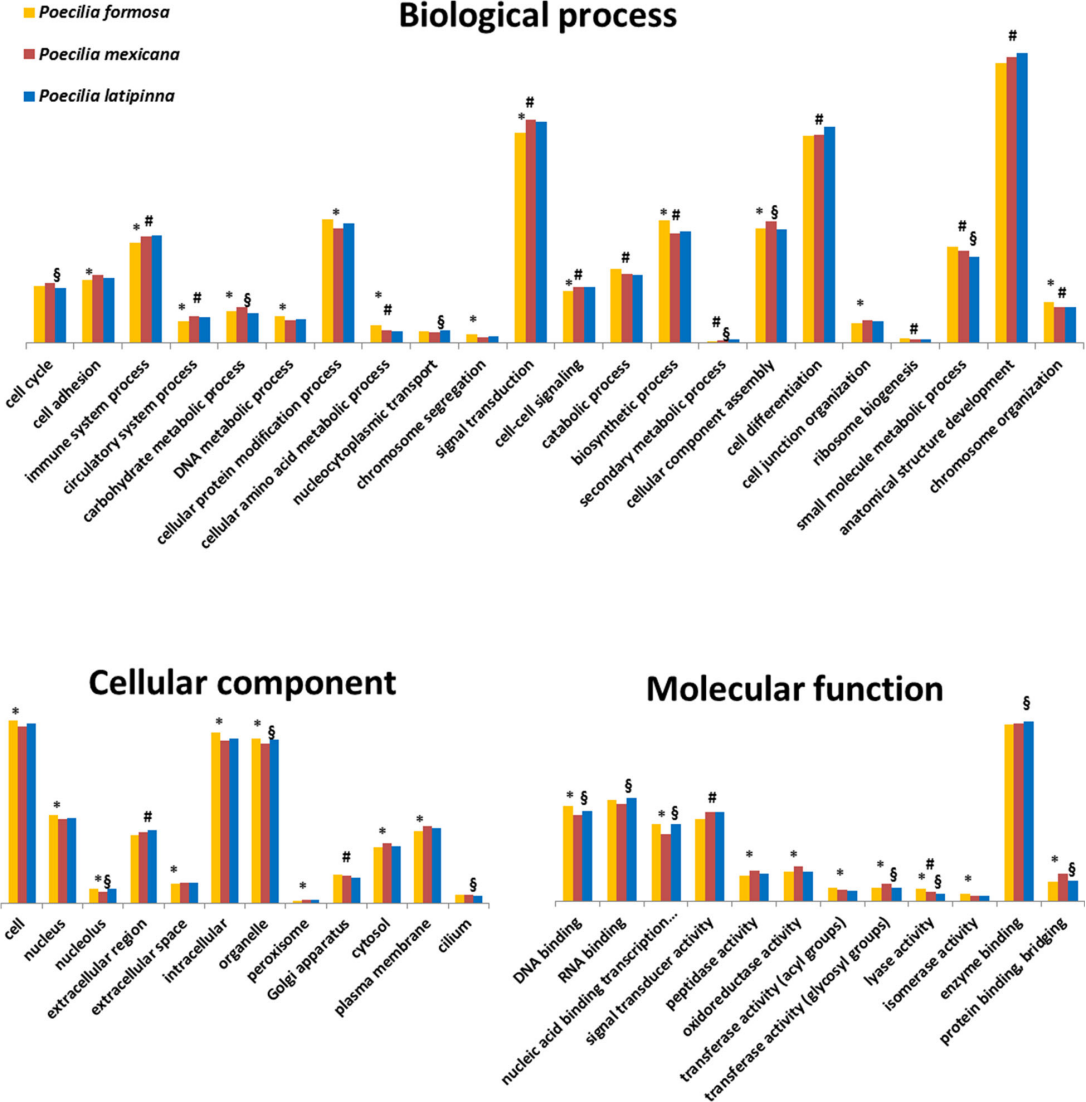
Occurrence of the represented generic GO slim terms (proportional to the total number) within the annotated transcriptomes of the Amazon molly (*P. formosa*) and its ancestral species *P. latipinna* and *P. mexicana* for the three main categories “Biological process”, “Cellular component” and “Molecular function”. Significant differences (one sided Fisher-Test; *p* < 0.05 corrected for multiple testing via Benjamini-Hochberg) between the species are labelled as * for *P. formosa/P. mexicana*; **#** for *P. formosa/P. latipinna* and **§** for *P. latipinna/P. mexicana*

Functional gene annotation with the GOblet software yielded 47,719 transcripts assigned to GO terms for *P. formosa* (44.63%), 46,157 (40.08%) for *P. latipinna*, and 55,659 (41.84%) for *P. mexicana*, based on sequence similarity comparisons with the UniProt/Swiss-Prot databases of vertebrates, rodents, human, and mammals (total entries: 47,483). In total, 19,227 different GO terms were detected among all three transcriptomes; 18,531 of these were shared between all three species (total number of GO terms for *P. formosa*: 18,856 | *P. latipinna*: 18,807 | *P. mexicana*: 19,019); 85 GO terms are unique for the *P. formosa* (70 transcripts) assembly (Fig. 1). The relative frequency of found generic GO slim terms was similar for all three species. Significant differences for the GO terms enrichment analysis between the species could be found for 32 GO terms between *P. formosa* and *P. mexicana* and for 17 GO terms between *P. formosa* and *P. latipinna* (Fig. 2), six of which were significantly different to the unisexual *P. formosa* in both sexual species (i.e., “immune system process”, “cellular amino acid metabolic process”, “signal transduction”, “cell-cell-signalling”, “biosynthetic process”, and “lyase activity”). The GO enrichment analysis for the detected GO terms for each species (Additional files 3, 4 and 5: Figures S2, S3, S4) did not reveal any differences among the three species.

Sequence comparisons among teleost fish included our three species, two additional species from the family Poeciliidae, *i*.*e*., the guppy (*P. reticulata*) and the common platy (*Xiphophorus maculatus*), and two well-annotated model organisms, the Japanese medaka *(Oryzias latipes*) and the zebrafish (*D. rerio*) (Table 3). In comparison with the two model species, the three assemblies showed similar results. *P. formosa* had a slightly higher congruency with the entire Swiss-Prot database (52.45%) in comparison with *P. latipinna* and *P. mexicana* (46.48% and 48.54%, respectively). All BLAST results were scanned for 108 meiosis-related genes obtained from the literature and databases, 46 of these genes are specific to the meiotic cell cycle (Table 4). Three common housekeeping genes [60, 61], the gene for the TATA-box binding protein (*tbp*), the hypoxanthine phosphoribosyl transferase 1 (*hprt1*), and Beta-actin (*actb*), were inspected and found to be equally present in all three species in terms of the number of counts. Out of the 108 meiosis-related genes, only the *stra8* gene and the meiosis-specific *hormad2* gene could not be detected in any of the assemblies of our study species. Four other genes were not found for *P. formosa*: the *ccnB1ip* gene, the *xycp1* gene and two meiosis-specific genes (*rad51B* and *rec114*). In total 1335 transcript counts of meiosis-related genes were discovered for *P. formosa,* markedly fewer than the 2313 counts for *P. mexicana* and 2054 for *P. latipinna*.

**Table 3:** Summary of BLAST comparisons

Taxa		Entries	BLAST algorithm	*Poecilia formosa*	*Poecilia latipinna*	*Poecilia mexicana*
*Poecilia formosa*	cDNA	30,958	tblastx	67,957 (63.56%)	63,547 (55.17%)	74,800 (56.23%)
	DNA	3985	blastn	106,300 (99.4 2%)	114,516 (99.43%)	131,651 (98.97%)
	protein	30,898	blastx	49,267 (46.08%)	48,170 (41.82%)	58,574 (44.03%)
*Poecilia mexicana*	DNA	18,105	blastn	106,151(99.28%)	113,876 (98.87%)	131,866 (99.13%)
	protein	47,406	blastx	51,032 (47.73%)	49,608 (43.07%)	60,442 (45.44%)
*Poecilia latipinna*	DNA	17,988	blastn	106,127 (99.26%)	114,721 (99.61%)	130,691 (98.25%)
	protein	47,072	blastx	51,096 (47.79%)	49,757 (43.20%)	60,227 (45.27%)
*Poecilia reticulata*	DNA	43,715	blastn	49,894 (47.73%)	48,762 (42.34%)	59,326 (44.60%)
	protein	2768	blastx	100,357 (93.86%)	107,025 (92.92%)	122,599 (92.13%)
*Xiphophorus maculatus*	cDNA	20,482	tblastx	67,957 (63.56%)	63,547 (55.17%)	74,800 (56.23%)
	DNA	20,632	blastn	106,300 (99.42%)	114,516 (99.43%)	131,651 (98.97%)
	protein	20,454	blastx	49,267 (46.08%)	48,170 (41.82%)	58,574 (44.03%)
*Oryzias latipes*	cDNA	24,675	tblastx	39,246 (36.71%)	40,351 (35.03%)	49,354 (37.10%)
	DNA	7189	blastn	15,343 (14.35%)	16,805 (14.59%)	21,320 (16.03%)
	protein	24,674	blastx	38,139 (35.67%)	38,839 (33.72%)	47,559 (35.75%)
*Danio rerio*	cDNA	48,435	tblastx	37,939 (35.48%)	39,132 (33.98%)	47,942 (36.04%)
	DNA	1133	blastn	2155 (2.02%)	3066 (2.66%)	4005 (3.01%)
	protein	43,153	blastx	37,216 (34.81%)	38,463 (33.40%)	46,709 (35.11%)
Swiss-Prot	protein	547,964	blastx	56,085 (52.45%)	53,528 (46.48%)	64,569 (48.54%)

For each taxon, we show the number of the sequences of the cDNA/DNA (toplevel) and protein databases, the BLAST algorithm used, and the percentage of matched sequences. cDNA resources were utilized when available for the respective species

**Table 4:** Genes associated with meiosis, their Uniprot accession ID and the number of the corresponding transcripts in the Amazon molly (*P. formosa:* Pfor), the Sailfin molly (*P. latipinna:* Plat) and Atlantic molly (*P. mexicana:* Pmex) transcriptomes

Gene	Description	Accession number	Number of transcripts		
			Pfor	Pmex	Plat
*ago1*	Argonaute 1, Eukaryotic translation initiation factor 2C 1	Q8CJG1	7	13	10
*ago2*	Argonaute 2, Eukaryotic translation initiation factor 2C 2	Q8CJG0	12	24	20
*ago3*	Argonaute 3, Eukaryotic translation initiation factor 2C 3	Q9H9G7	31	38	55
*ago4*	Argonaute 4, Eukaryotic translation initiation factor 2C 4	Q9HCK5	20	16	21
*ccnA1*	Cyclin-A1	Q92161	4	2	9
*ccnA2*	Cyclin-A2	P30274	3	4	6
*ccnB1ip1*	Cyclin B1 interacting protein 1	Q9NPC3	0	1	1
*ccnC*	Cyclin-C	Q28F72	3	3	5
*cdk1*	Cell division protein kinase/Cyclin-dependent kinase 1	Q9DG98	38	73	52
*cdk2*	Cell division protein kinase/Cyclin-dependent kinase 2	P43450	8	23	5
*cdk4*	Cyclin-dependent kinase 4	Q91727	12	3	5
*cdk7*	Cyclin-dependent kinase 7	P51953	4	2	3
*cdk10*	Cell division protein kinase/Cyclin-dependent kinase 10	Q2TBL8	4	7	5
*cdk14*	Cell division protein kinase/Cyclin-dependent kinase 14	B0VXL7	59	173	110
*cdk16*	Cell division protein kinase/Cyclin-dependent kinase 16	Q00536	19	19	9
dmc1*	Meiotic recombination protein DMC1	Q61880	1	3	2
fkbp6*	Inactive peptidyl-prolyl cis-trans isomerase FKBP6	Q91XW8	1	1	2
*hfm1*	Probable ATP-dependent DNA helicase HFM1	A2PYH4	14	41	27
hormad1*	HORMA domain-containing protein 1	Q86X24	3	2	2
hormad2*	HORMA domain-containing protein 2	Q8N7B1	0	0	0
*m1ap*	Meiosis 1 arrest protein	Q9Z0E1	67	44	65
majin*	Membrane-anchored junction protein	Q9D992	11	28	18
*marf1*	Meiosis arrest female protein 1	Q8BJ34	16	29	12
*mcm2*	DNA helicase MCM2, Minichromosome maintenance protein 2	Q6DIH3	13	29	8
*mcm3*	DNA helicase MCM3, Minichromosome maintenance protein 3	Q5ZMN2	3	9	11
*mcm4*	DNA helicase MCM4, Minichromosome maintenance protein 4	P33991	2	2	4
*mcm4B*	Minichromosome maintenance protein 4-B	P30664	1	1	2
*mcm5*	DNA helicase MCM5, Minichromosome maintenance 5	Q561P5	9	20	9
*mcm6*	DNA helicase MCM6, Minichromosome maintenance 6	Q14566	4	10	11
*mcm7*	DNA helicase MCM7, Minichromosome maintenance 7	Q6NX31	1	2	1
*mcm8*	DNA helicase MCM8, Minichromosome maintenance 8	Q9UJA3	20	74	21
*mcm9*	DNA helicase MCM9, Minichromosome maintenance 9	Q6NRM6	16	50	14
mei1*	Meiosis inhibitor protein 1	Q5TIA1	28	55	64
mei4*	Meiotic double-stranded break formation protein 4	Q8BRM6	17	41	56
meiob*	Meiosis-specific with OB domain-containing protein	Q9D513	6	4	15
meioc*	Meiosis-specific coiled-coil domain-containing protein	A2AG06	3	4	5
mlh1*	DNA mismatch repair protein Mlh1, MutL protein homolog 1	P40692	11	15	10
mlh3*	DNA mismatch repair protein Mlh3, MutL protein homolog 3	Q9UHC1	9	14	6
mnd1*	Meiotic nuclear division protein 1 homolog	Q32L19	40	183	80
mns1*	Meiosis-specific nuclear structural protein 1	Q6PBA8	6	6	4
*mre11*	Double-strand break repair protein MRE11	Q9W6K1	9	6	6
msh2*	DNA mismatch repair protein Msh2, MutS protein homolog 2	Q5XXB5	18	29	31
msh3*	DNA mismatch repair protein Msh3, MutS protein homolog 3	P20585	15	37	33
msh4*	DNA mismatch repair protein Msh4, MutS protein homolog 4	O15457	5	1	6
msh5*	DNA mismatch repair protein Msh5, MutS protein homolog 5	O43196	6	8	6
msh6*	DNA mismatch repair protein Msh6, MutS protein homolog 6	P52701	5	7	4
*nbn*	Nibrin	O60934	6	9	4
*piwil1*	Piwi-like protein 1	Q8UVX0	5	2	4
*piwil2*	Piwi-like protein 2	A6P7L8	20	27	19
*pms1*	PMS1 protein homolog 1	P54277	32	51	22
*pms2*	DNA mismatch repair protein (endonuclease) PMS2	P54278	6	4	5
*prdm9*	Histone-lysine N-methyltransferase	Q96EQ9	5	6	7
psmc3ip*	Homologous-pairing protein 2 homolog (HOP2)	Q63ZL2	2	1	1
*rad1*	Cell cycle checkpoint protein RAD1	Q5R7X9	9	4	3
rad21*	Double-strand-break repair protein rad21 homolog	O60216	34	39	33
*rad50*	DNA repair protein RAD50	P70388	3	5	4
*rad51*	DNA repair protein RAD51 homolog 1	Q06609	5	2	3
rad51B*	DNA repair protein RAD51 homolog 2/B	Q91917	0	15	3
rad51C*	DNA repair protein RAD51 homolog 3/C	O43502	2	1	1
rad51D*	DNA repair protein RAD51 homolog 4/D	O75771	31	27	189
*rad52*	DNA repair protein RAD52 homolog	P39022	1	3	1
rad54A*	DNA repair and recombination protein RAD54-like	Q92698	5	12	10
rad54B*	DNA repair and recombination protein RAD54B	Q9DG67	20	19	28
*rad9A*	Cell cycle checkpoint control protein RAD9A	Q99638	4	6	8
*rad9B*	Cell cycle checkpoint control protein RAD9B	Q6WBX8	7	20	25
rec8*	Meiotic recombination protein REC8	O95072	20	39	180
rec114*	Meiotic recombination protein REC114	Q7Z4M0	0	2	2
*recQl1*	ATP-dependent DNA helicase Q1	Q9Z129	30	115	82
*recQl4*	ATP-dependent DNA helicase Q4	O94761	78	49	63
*recQl5*	ATP-dependent DNA helicase Q5	O94762	9	8	6
*rmi1*	RecQ-mediated genome instability protein 1	A4IF98	3	2	2
*rmi2*	RecQ-mediated genome instability protein 2	Q5ZM20	4	2	2
*rnf212*	Ring finger protein 212 / Probable E3 SUMO-protein ligase	F6TQD1	11	9	6
sfr1*	SWI5 Dependent Homologous Recombination Repair Protein 1	B7ZD04	4	1	1
*sgo2*	Shugoshin 2	Q7TSY8	9	5	11
*smarca2*	SWI/SNF-related matrix-associated actin-dependent regulator of chromatin subfamily A member 2	Q6DIC0	44	80	77
*smarca4*	SWI/SNF-related matrix-associated actin-dependent regulator of chromatin subfamily A member 4	A7Z019	17	29	36
smc1a*	Structural maintenance of chromosomes protein 1A	Q9CU62	6	4	4
smc1b*	Structural maintenance of chromosomes protein 1B	Q8NDV3	10	75	17
smc2*	Structural maintenance of chromosomes protein 2	P50533	5	8	4
smc3*	Structural maintenance of chromosomes protein 3	Q9CW03	29	47	18
smc4*	Structural maintenance of chromosomes protein 4	P50532	9	8	8
smc5*	Structural maintenance of chromosomes protein 5	Q802R9	3	1	4
smc6*	Structural maintenance of chromosomes protein 6	Q6P9I7	36	54	48
*smchd1*	Structural maintenance of chromosomes flexible hinge domain-containing protein 1	A6NHR9	10	19	24
*spdya-A*	Speedy protein 1-A	Q9PU13	3	1	5
spo11*	Meiotic recombination protein	Q9Y5K1	5	18	3
stag1*	Cohesin subunit SA-1	Q8WVM7	18	45	36
stag2*	Cohesin subunit SA-2	Q8N3U4	18	27	19
stag3*	Cohesin subunit SA-3	O70576	19	12	24
*stra8*	Stimulated by retinoic acid gene 8 protein	P70278	0	0	0
*syce1*	Synaptonemal complex central element protein 1	Q8N0S2	1	1	1
syce2*	Synaptonemal complex central element protein 2	Q505B8	10	6	8
syce3*	Synaptonemal complex central element protein 3	B5KM66	1	2	1
*sycp1*	Synaptonemal complex protein 1	Q62209	0	4	6
*sycp2*	Synaptonemal complex protein 2	Q9CUU3	8	8	6
*sycp3*	Synaptonemal complex protein 3	P70281	6	15	2
terb1*	Telomere repeats-binding bouquet formation protein 1	Q8NA31	5	3	3
terb2*	Telomere repeats-binding bouquet formation protein 2	Q9D494	2	1	1
tex11*	Testis-expressed sequence 11 protein	Q8IYF3	43	53	46
*TOP6BL*	Type 2 DNA topoisomerase 6 subunit B-like	Q8N6T0	18	43	9
*trip13*	Pachytene checkpoint protein 2 homolog	Q15645	5	2	7
*wWee2*	Wee1-like protein kinase 2	Q66JT0	10	36	12
*xrcc1*	DNA repair protein XRCC1	Q60596	3	2	2
xrcc2*	DNA repair protein XRCC2	Q9CX47	9	8	21
xrcc3*	DNA repair protein XRCC3	Q08DH8	4	3	2
*xrcc4*	DNA repair protein XRCC4	Q924T3	27	52	23
*zmcm3*	Zygotic minichromosome maintenance protein 3	Q7ZXZ0	2	5	1

Genes specific for meiosis are labeled with an asterisk (*)

**Fig. 3:**
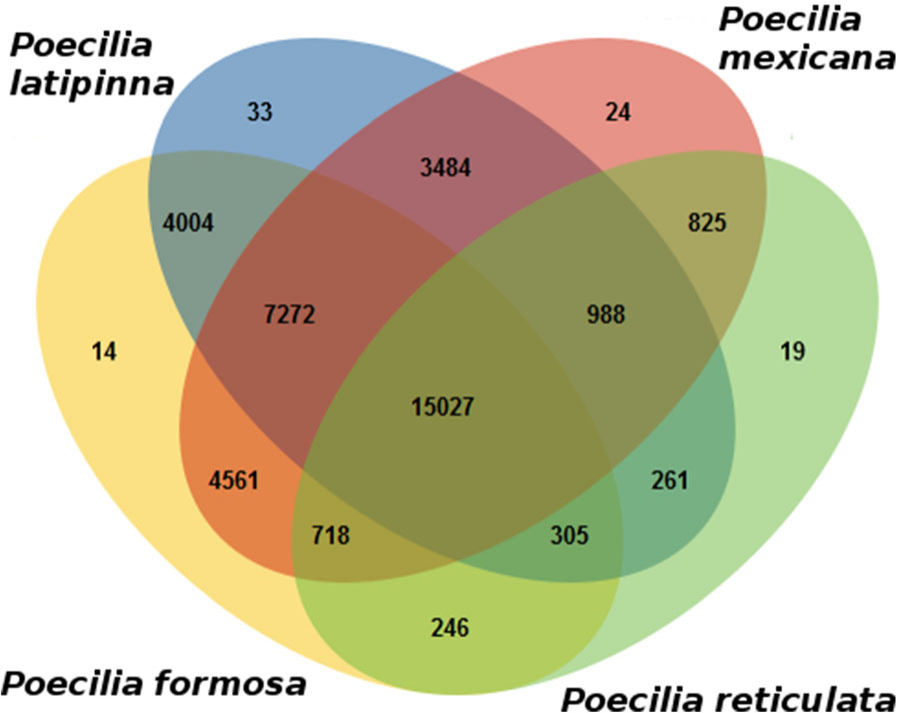
Identified orthogroups for the transcriptomes of *P. formosa*, *P. mexicana*, and *P. latipinna* in comparison with the proteome of *P. reticulata*

**Fig. 4:**
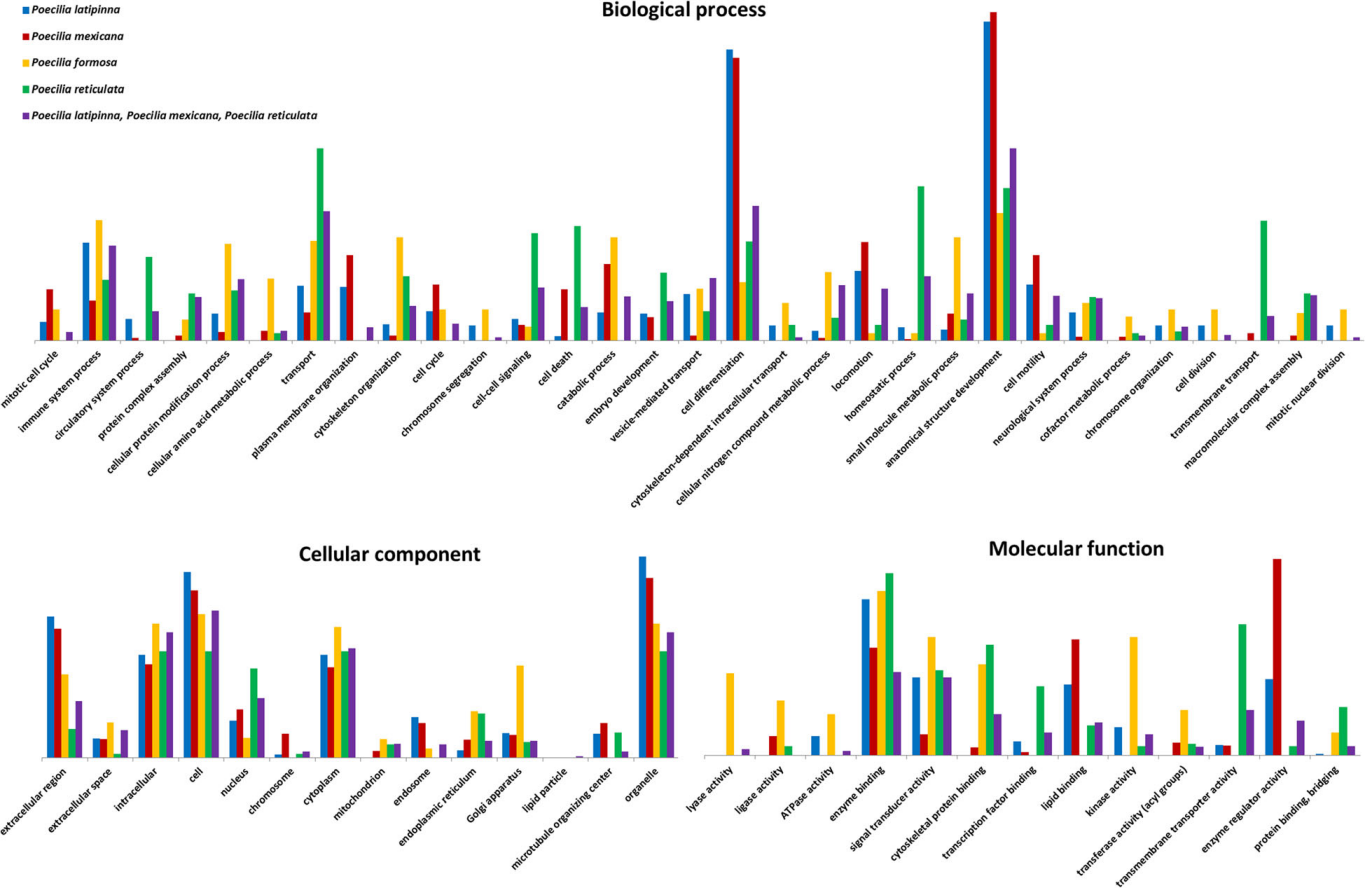
Number of unique gene ontology term entities for the annotated orthogroups for *P. formosa, P. mexicana, P. latipinna, P. reticulata* and the sexual species in combination (*P. latipinna, P. mexicana, and P. reticulata*). Only generic slim GO terms significantly different in their occurrence (one-sided Fisher-Tests; *p* < 0.05 corrected for multiple testing via Benjamini-Hochberg), between *P. formosa* and the other species are shown; bar sizes are proportional to their total number of occurrences

To identify putative orthologues, the transcripts were first analyzed by the Transdecoder pipeline, beginning by translating the contigs into amino acid sequences. The total number of ORFs regardless to their coding potential was 218,390 amino acid sequences for *P. formosa*, 251,006 for *P. latipinna*, and 318,099 for *P. mexicana*. All amino acid sequences were compared via the blastp algorithm to the UniProt/Swiss-Prot database, yielding 44,860 (20.54%) matches for *P. formosa*, 57,563 (22.93%) for *P. latipinna*, and 73,013 (22.95%) for *P. mexicana*. Homology comparisons with the pfam database resulted in 72,519 matches for *P. formosa* (corresponding to 13,341 unique database entries), 78,797 for *P. latipinna* (corresponding to 13,388 unique database entries), and 99,659 for *P. mexicana* (corresponding to 13,603 unique database entries). In total, the Transdecoder analysis yielded 82,815 amino acid sequences predicted as likely coding regions for *P. formosa*, 87,235 for *P. latipinna*, and 109,824 for *P. mexicana*, which were all fed into the OrthoFinder pipeline, together with the *P. reticulata* proteome (Table 5). For the 323,589 amino acid sequences across all four species, 77.24% were assigned to 37,781 orthogroups with a median group size of four genes. An orthogroup includes the orthologous genes of the compared species and is defined as the group of genes descended from a single gene in the last common ancestor of a group of species. 74.38% of the amino acid sequences of *P. formosa* were assigned to orthogroups with *P. reticulata* (*P. mexicana*: 69.45% | *P. latipinna*: 73.42%). All four species shared 15,027 orthogroups. Ninety orthogroups (comprising 1052 genes, corresponding to 0.33% of all genes) were species-specific, i.e., they consisted entirely of genes detected only in one species. Specifically, 14 orthogroups were unique for *P. formosa*, 33 for *P. latipinna*, 24 for *P. mexicana*, and 19 for *P. reticulata* (Fig. 3, created with the online application jvenn [62]). The unique orthogroups for each of the four species and the 988 orthogroups, which were exclusively identified among the three sexual species, were annotated to detect differences in the occurrence of the corresponding GO terms (generic slim) between the sexual and the unisexual species (Fig. 4). In the sexual species, there are more genes annotated to the GO term “embryo development” (GO:0009790) or “chromosome” (GO:0005694) than in the unisexual *P. formosa*. In contrast, *P. formosa* exhibits more genes in unique orthogroups for different enzyme activities (for example, “ligase activity” (GO:0016874)). None of the orthogroups specific for *P. formosa* was associated with reproduction or meiosis. The analysis of the 988 orthogroups shared among the sexual species revealed 34 additional genes related to the meiotic cell cycle (Additional files 6: Table S2, Additional files 7, 8 and 9).

**Table 5:** Orthology analysis using OrthoFinder

	*Poecilia formosa*	*Poecilia latipinna*	*Poecilia mexicana*	*Poecilia reticulata*
Total number of genes	82,815	87,235	109,824	43,715
Number of genes in orthogroups (%)	61,651 (74.44%)	65,588 (75.19%)	82,547 (75.16%)	40,138 (91.18%)
Number of unassigned genes	21,164	21,647	27,277	3577
Number of orthogroups (%)	32,147 (85.09%)	31,374 (83.04%)	32,899 (87.08%)	18,389 (48.67%)
Number of species-specific orthogroups (genes)	14 (129)	33 (385)	24 (370)	19 (168)

**Fig. 5:**
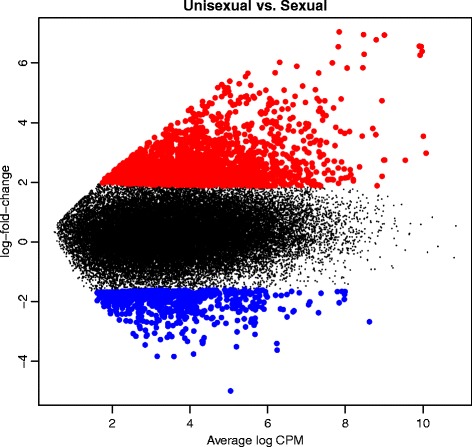
Mean difference plot showing the log-fold change and average abundance of each gene of the differential expression analysis between *P. formosa* (“Unisexual”) and *P. mexicana* and *P. latipinna* (“Sexual”). Color depicts genes down-regulated (blue) or up-regulated (red) in *P. formosa*

**Fig. 6:**
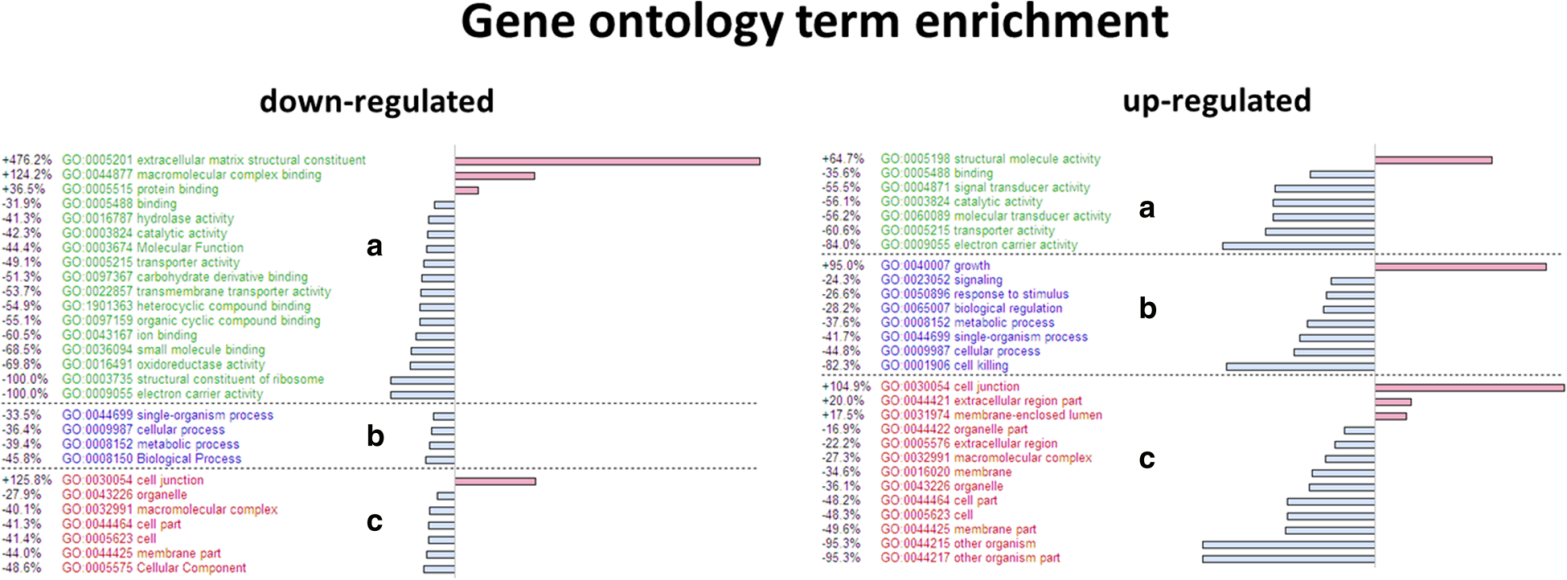
Gene ontology term enrichment for the differentially expressed genes between the unisexual (*P. formosa*) and sexual species (*P. mexicana* and *P. latipinna*) generated by GOblet. Only significantly enriched (red) or depleted (blue) generic GO terms are shown (one-sided Fisher-Tests; *p* < 0.05), for the three components “Molecular function (**a**)”, “Biological process (**b**)” and “Cellular components” (**c**)

Compared to its sexually reproducing parental species, 2035 (4.69%) genes were up-regulated and 564 (1.30%) genes were down-regulated in the unisexual *P. formosa* identified at a false discovery rate (FDR) of 5% for the 43,356 tested genes, corrected via the Benjamini and Hochberg’s algorithm (Fig. 5). The differentially expressed genes associated with the GO terms “reproduction” (GO:000003) and “reproductive process” (GO:0022414) are listed in Table 6. Twenty seven genes related to reproduction have a higher expression in *P. formosa*, e.g., the gene of the Speedy protein A. For the GO enrichment of the GO term “cell junction” (GO:0030054), up- and down-regulated genes were over-represented. This means some genes of this GO term may be up-regulated in the sexual, others in the unisexual species. This is indicative of an alteration of gene activity between the unisexual and sexual species (Fig. 6). We exemplary compared our expression patterns to gene-specific expression data for six genes of the androgen receptor pathway by qRT-PCR on the same RNA isolates used for the transcriptome analysis (data from [33]). One gene (*cyp19a2*) was consistently up-regulated in the asexual species (1.9 fold in qRT-PCR; 1.4 fold with regard to transcriptome read numbers). Two genes (*erα* and *erβ*) were consistently up-regulated in the sexual species (1.7 resp. 1.6 fold in the qRT-PCR; both 1.9 fold in the transcriptome analysis). Two further genes (*arβ* and *cyp19a2*) were not differentially expressed in neither the qRT-PCR study nor the transcriptome analysis. For one gene (*arα*), the transcriptome data exhibited a 3.7 fold higher read number in the asexual species, relative to the sexual species. This was not confirmed by qRT-PCR, but expression at this gene was very variable among six biological replicates in one of the sexual species, *P. latipinna* (2.9 fold within 95% confidence limits). While the up-regulation of *arα* detected in the transcriptome data for the asexual species may be hence a false positive (presumably caused by the variable expression in one of the sexual species), we find overall consistent expression patterns in five (out of six) analyzed genes among transcriptome read number analysis and a gene-specific qRT-PCR analysis. The scale of expression differences (fold change) was also similar among the two methods.

**Table 6:** Detected GO term IDs, the GO term names and the corresponding genes for the up-regulated (+) or down-regulated (−) in *P. formosa* (only genes involved in reproduction and meiosis are listed)

GO term ID	GO term name	Gene	Description	Expression
GO:0000212	Meiotic spindle organization	*larp*	La-related protein 1	(+)
		*tubgcp4*	Gamma-tubulin complex component 4	(+)
GO:0000706	Meiotic DNA double-strand break processing	*atr*	Serine/threonine-protein kinase ATR	(+)
GO:0000710	Meiotic mismatch repair	*xpc*	DNA repair protein complementing XP-C cells	(+)
GO:0000711	Meiotic DNA repair synthesis	*ccng1*	Cyclin-G1	(+)
		*ccng2*	Cyclin-G2	(+)
GO:0001555	Oocyte growth	*rbp4a*	Retinol-binding protein 4-A	(+)
		*rbp4b*	Retinol-binding protein 4-B	(+)
GO:0007111	Meiosis II cytokinesis	*actba*	Actin, cytoplasmic 1	(+)
GO:0007130	Synaptonemal complex assembly	*bag6*	Large proline-rich protein BAG6;	(+)
GO:0007131	Reciprocal meiotic recombination	*topbp1-A*	DNA topoisomerase 2-binding protein 1-A	(+)
GO:0007286	Spermatid development	*abhd2-A*	Monoacylglycerol lipase ABHD2-A	(+)
GO:0007288	Sperm axoneme assembly	*neurl1*	E3 ubiquitin-protein ligase NEURL1	(+)
GO:0008584	Male gonad development	*acvr2A*	Activin receptor type-2A	(−)
		*ncoa1*	Nuclear receptor coactivator 1	(+)
GO:0016344	Meiotic chromosome movement towards spindle pole	*fmn2*	Formin-2	(+)
GO:0019102	Male somatic sex determination	*ar*	Androgen receptor	(+)
GO:0040022	Feminization of hermaphroditic germline	*dhx16*	Putative pre-mRNA-splicing factor ATP-dependent RNA helicase DHX16	(+)
GO:0044779	Meiotic spindle checkpoint	*ttk*	Dual specificity protein kinase Ttk	(+)
GO:0045141	Meiotic telomere clustering	*sun1*	SUN domain-containing protein 1	(+)
GO:0048477	Oogenesis	*lrmp*	Lymphoid-restricted membrane protein	(−)
GO:0051039	Positive regulation of transcription involved in meiotic cell cycle	*brd2*	Bromodomain-containing protein 2	(+)
GO:0051177	Meiotic sister chromatid cohesion	*anchr*	Abscission/NoCut checkpoint regulator	(+)
GO:0051307	Meiotic chromosome separation	*mcm5A*	DNA replication licensing factor mcm5-A	(−)
GO:0051446	Positive regulation of meiotic cell cycle	*spdya*	Speedy protein A	(+)
GO:0051447	Negative regulation of meiotic cell cycle	*dusp1*	Dual specificity protein phosphatase 1	(+)
GO:0051598	Meiotic recombination checkpoint	*rad1*	Cell cycle checkpoint protein RAD1	(+)
GO:0090306	Spindle assembly involved in meiosis	*aspm*	Abnormal spindle-like microcephaly-associated protein	(+)
GO:1,903,537	Meiotic cell cycle process involved in oocyte maturation	*pgrmc1*	Membrane-associated progesterone receptor	(+)
GO:1,903,538	Regulation of meiotic cell cycle process involved in oocyte maturation	*prkar1a*	cAMP-dependent protein kinase type I-alpha regulatory subunit	(+)

## Discussion

### Quality of the de novo transcriptome assemblies

De novo assembly of the datasets resulted in a higher number of transcripts for the Atlantic molly *P. mexicana* and the sailfin molly *P. latipinna*, compared to the Amazon molly *P. formosa* and sequencing statistics were overall quite similar between the parental species, especially regarding the N50 value and the average contig length. On average, the statistics for the de novo assemblies show similar results compared to other transcriptomes of fish species using RNA sequencing techniques (Illumina) [63–66]. The higher number of total transcripts for all three species compared to other transcriptomes of the family Poeciliidae, for example, the *P. mexicana* transcriptome (number of transcripts: 80,111) [63] or the transcriptome of the Western mosquitofish *Gambusia affinis* (average number of transcripts: 63,734) [64], can be likely attributed to the fact that we retained some of the transcripts with a low expression, which some other authors may have filtered out. We – on purpose – retained these transcripts in order to maintain our ability to detect genes expressed in a species-specific manner.

For the Trinity assembler, which is well suited for the reconstruction of transcriptomes de novo [38, 67, 68], each component (also referred to as unigenes) represents a set of transcripts, which are assumed to represent genes (*P. formosa*: 59,935 | *P. latipinna*: 73,450 | *P. mexicana*: 79,522) and include different isoforms (transcripts) derived from alternative splicing or closely related paralogs. Based on the longest isoform for each component, all three assemblies are more similar in the N50 value (*P. formosa*: 1510 bp | *P. latipinna*: 1654 bp | *P. mexicana*: 1726 bp) and the average contig length (*P. formosa*: 865 bp | *P. latipinna*: 843 bp | *P. mexicana*: 859 bp). Comparing our transcriptomes to the annotation releases of the three *Poecilia* species genomes, *P. formosa* has a lower number of transcripts and components than both ancestral species; this appears to reflect the actual composition of the datasets. A lower number of genomic mRNA transcripts has been previously reported for *P. formosa* (39,207), compared to *P. mexicana* (47,406) and *P. latipinna* (47,072) (NCBI annotation release 100, 2015).

All three de novo assembled transcriptomes exhibited comparable quality measures in downstream analyses, like functional annotation and the comparative analysis of sequence similarities with the different teleost databases. Also, the ratio of transcripts without ORFs and possible contaminants for all assemblies was similar. The contamination load obtained was only between 1.6 and 1.7% of the transcripts per species. All three de novo assemblies showed high consistency with the different genomic and proteomic datasets. By implication, the agreement with the closer related species of the Poeciliidae family was higher in comparison to the less closely related species, like the Japanese medaka *O. latipes* or the zebrafish *D. rerio*. Even with very strict mapping parameters, a high percentage of reads mapped back to the transcripts (*P. formosa*: 76.39% | *P. latipinna*: 78.69% | *P. mexicana*: 77.63%), which matches the desired range (between 70 and 80%) described in the Trinity user guide. In summary, overall results are similar for all the de novo assembly datasets, suggesting that the transcriptomes for all three species were suitable for comparative analysis.

### Differential gene expression between unisexual and bisexual mollies

Based on the clustering and count data of the mapped reads of the species with themselves and between them, we performed a differential expression analysis comparing two conditions (unisexual *vs*. sexual). We considered the two sexual species as biological replicates and compared this group to the unisexual species. As a second related unisexual species does not exist, we do not have a species replicate for the unisexual condition. We were hence unable to establish statistical significance for the inferred 2035 up-regulated and 564 down-regulated genes identified for the unisexual *P. formosa*. We exemplary confirmed the transcriptome-derived expression patterns in five (out of six) genes analyzed by qRT-PCR. We are also aware of that an unknown number of differentially expressed genes may have gone undetected and a thorough analysis of differential expression would require a higher number of replicates per condition [69]. Nonetheless, we argue – with caution – that differences in read numbers in our transcriptome data may have revealed candidates for genes differentially expressed among sexual *vs*. unisexual species, to be further analyzed in future research.

We used three different approaches to identify candidate genes, which may be involved in the molecular underpinning of the different reproduction modes among the sexual and unisexual species. First, we searched the BLAST results for the occurrence of genes related to meiosis or reproduction. Second, we conducted an orthology analysis with a closely related species, the guppy *P. reticulata.* Finally, as described above, we identified differentially expressed genes, *i*.*e*. those, which are higher or lower expressed in *P. formosa*, as compared to its parental species. Scanning the BLAST results for the occurrence of 108 meiosis-related genes showed that 1.25% (equates to 1335 transcripts) of all generated transcripts for *P. formosa* are linked to the meiotic cell cycle which is significantly lower compared to 1.74% for *P. mexicana* and 1.78% for *P. latipinna* (*p* < 0.05 in both pairwise comparisons, tested with χ^2^ test). The ratio of the meiosis-specific genes to the total number of transcripts is 0.50% in *P. formosa* (*P. mexicana*: 0.73% | *P. latipinna*: 0.92%). In line with the lack of meiosis in *P. formosa*, a significantly lower percentage of transcripts was related to this process, in comparison to the sexual species. Yet, the down-regulation of meiosis-related genes is not as complete as one might have expected for a species producing gametes apomictically. Only two meiosis-related genes could not be detected in any of the three transcriptomes (*str8* and *hormad2*). The stimulated by retinoic acid gene 8 (*str8*) is required in mice for the transition of female and male germ cells into meiosis and is typically expressed in adult testes and embryonic ovaries [70]. Therefore, this gene is not necessarily expressed in adult female gonads, the tissue analyzed here. The second absent gene was the *hormad2* gene, which encodes the HORMA domain-containing 2 protein. The *hormad1* and *hormad2* genes are explicitly expressed during meiosis in male and female mice [71], but nothing is known about their function in fish.

In *P. formosa*, the most prominent meiosis-specific gene lacking in the transcriptome was the gene for meiotic recombination protein Rec114, required for DNA double strand break (DSB) formations, which induces meiotic recombination [72]. Studies in mice showed that the *rec114* gene is expressed in adult testes and in embryonic ovaries and seems to be conserved among most sexually reproducing eukaryotes [73]. This gene was not found in a previously published transcriptome of the Amazon molly either [74]. The functional annotation of the homologous genes for *P. mexicana*, *P. latipinna*, and the closely related *P. reticulata* yielded 35 genes of interest, which were absent in the Amazon molly transcriptome. Particularly interesting is the gene for the ATP-dependent RNA helicase *cgh1* (conserved germline helicase-1). In the hermaphrodite *Caenorhabditis elegans*, it is responsible for regulating maternal mRNA translational repression and protecting it from degradation (reviewed in [75]). The absence of this gene in *C. elegans* and presumably in other organisms leads to non-functional sperm and, more importantly, to the degradation of developing oocytes [76].

### Evolutionary implications of lowered expression in meiosis-related genes

Our results raise questions about the function of the detected and missing genes expressed in the Amazon molly *P. formosa* gonads as well as about its reproduction mechanisms. The presence or absence of transcripts related to a specific process (in this case reproduction and especially meiosis) lead to expectations about their evolution in asexual species compared to sexual ones. If a certain biological process is no longer maintained, the underlying genes are expected to be under reduced functional constraints (relaxed selection), leading to the accumulation of deleterious mutations, which may compromise their biological function and/or their expression. Ultimately, genes may degenerate such that they can become pseudogenes [77, 78]. The time span since *P. formosa* evolved from its ancestor species (280,000 years [19]) may have been too short to result in pervasive pseudogenization of meiosis genes. Nonetheless, the generally lower expression levels and the lack of expression in several such genes, some of which of crucial importance in sexual reproduction, points to an evolutionary erosion of genes no longer necessary in an apomictic species.

However, meiosis genes are not always under relaxed selection in asexually reproducing species. In a comparison of obligate sexual and asexual individuals in the freshwater snail *Potamopyrgus antipodarum*, three meiosis-specific genes (*spo11*, *msh4* and *msh5*) exhibited no degeneration in the asexual lineages, but were instead inferred to be under purifying selection [79]. Also, for three ancient asexual oribatid mites, there is stronger purifying selection on nuclear and mitochondrial orthologous genes compared to sexual species [80]. For the microcrustacean *Daphnia pulex,* whose reproduction cycle consists of alternating sexual and asexual phases, the main meiosis genes are present in the genome and are expressed under parthenogenesis [9]. These genes could gain new or until now undiscovered functions, possibly leading to novel alternative pathways to meiosis. For example, the *spo11* gene – known to initiate meiotic recombination by the introduction of DSBs in sexual species – has been described to lead to extensive genetic recombination between homologous chromosomes, including multiple gene conversion events, in an ameiotic species, the parasexual fungus *Candida albicans* [81]. Gene conversion has been frequently detected in *P. formosa* [18], but deeper molecular knowledge is needed to unravel, whether there are potential alternate functions of meiosis genes in this species. Comparing meiosis-specific genes on the intron/exon level among the three species can be a first approach to analyze their functions and to detect selective constraints. An additional approach would be to study knockout/knockdown individuals in comparison with the wild type, which is a well-established and extensively used genetic technique to directly examine functional and phenotypic effects of candidate genes [82], particularly in the fish model organisms *D. rerio* (reviewed in [83]) and *O. latipes* [84] as well as for *Carassius gibelio* [85], which has multiple reproduction modes including sexual reproduction and unisexual gynogenesis [86]. The dataset published in this study forms an excellent basis for further investigations, including those described above or for single nucleotide polymorphism (SNP) detection, and qRT-PCR, ideally conducted in an allele-specific manner, to resolve the evolutionary questions raised. Furthermore, our dataset would be beneficial for the (re-)annotation of the genomes of all three species.

## Conclusions

The generated de novo gonadal transcriptomes of the Amazon molly *Poecilia formosa* and its parental species, the sailfin molly *P. latipinna* and the Atlantic molly *P. mexicana*, were functionally annotated and analyzed on the basis of sequence similarities between the species. They provide a valuable resource for questions concerning the reproductive mode of an asexual hybrid species in comparison to its sexual ancestor species. Interestingly, there are also vertebrate examples, where hybrid speciation leads to an automictic form of parthenogenesis. Here, meiosis and recombination are maintained (e.g., in whiptail lizards, [87]). In contrast, our ameiotic species lacks recombination and is hence a ‘frozen hybrid’ at all nuclear loci [18, 33, 88].

Inline with our *a* *priori* hypothesis, there was a general tendency towards lower expression of meiosis-related genes in the apomictic *P. formosa*. However, only a few of these genes were completely absent in the *P*. *formosa* transcriptome, while the remainder constitutes interesting candidates for further evolutionary studies, *e*.*g*., on potential neofunctionalization *vs*. pseudogenization. Furthermore, our dataset comprises a substantial addition to the already present genomic resources available for the family of Poeciliidae and can be used for future sequencing projects as well as for the annotation of the genome for all three species.

## Additional files

Additional file 1: Table S1. Databases for the BLAST sequence similarity comparisons. (DOCX 14 kb)

Additional file 2: Figure S1. Transcript length distribution for the de novo assemblies of the Amazon molly (*P. formosa*), the sailfin molly (*P. latipinna*), and the Atlantic molly (*P. mexicana*). (BMP 1741 kb)

Additional file 3: Figure S2. Enrichment analysis of the generic GO slim terms evaluated using one-sided Fisher-Tests for *P. formosa* The. residues are given relative to the expected value, shown are significantly enriched (red) or depleted (blue) (*p* < 0,05) GOs for the three components: Molecular function (A), biological process (B), and cellular component (C). (BMP 4123 kb)

Additional file 4: Figure S3. Enrichment analysis of the generic GO slim terms evaluated using one-sided Fisher-Tests for *P. latipinna.* The residues are given relative to the expected value, shown are significantly enriched (red) or depleted (blue) (p < 0,05) GOs for the three components: Molecular function (A), biological process (B), and cellular component (C). (BMP 4278 kb)

Additional file 5: Figure S4. Enrichment analysis of the generic GO slim terms evaluated using one-sided Fisher-Tests for *P. mexicana.* The residues are given relative to the expected value, shown are significantly enriched (red) or depleted (blue) (p < 0,05) GOs for the three components: Molecular function (A), biological process (B), and cellular component (C). (BMP 4395 kb)

Additional file 6: Table S2. GO terms ID, the GO term names and the corresponding genes related to reproduction and meiosis for the orthogroups only detected in the sexual species. (DOCX 17 kb)

Additional file 7: Blast results for the sequence comparisons between the *Poecilia formosa* transcriptome and the Uniprot/Swiss-Prot database. (TXT 7495 kb)

Additional file 8: Blast results for the sequence comparisons between the *Poecilia latipinna* transcriptome and the Uniprot/Swiss-Prot database. (TXT 7176 kb)

Additional file 9: Blast results for the sequence comparisons between the *Poecilia mexicana* transcriptome and the Uniprot/Swiss-Prot database. (TXT 8636 kb)

## Abbreviations

BLAST: Basic local alignment search tool
cDNA: Complementary desoxyribonucleic acid
DSB: Double strand break
FDR: False discovery rate
GO: Gene ontology
GUI: Graphical user interface
IEA: Inferred from electronic annotation
N50 value: Weighted median length of transcripts
NCBI: National Center for Biotechnology Information
ORF: Open reading frame
qRT-PCR: Quantitative real-time Reverse Transcriptase Polymerase Chain Reaction
rRNA: ribosomal ribonucleic acid
SNP: Single nucleotide polymorphism
