# Supplementary material for: The gonadal transcriptome of the unisexual Amazon molly Poecilia formosa in comparison to its sexual ancestors, Poecilia mexicana and Poecilia latipinna
Source: BMC Genomics. 2018 Jan 3;19:12. doi: 10.1186/s12864-017-4382-2 (PMC5753479; doi:10.1186/s12864-017-4382-2)
Supplement: Supplementary file 1 — Databases for the BLAST sequence similarity comparisons. (DOCX 14 kb) [file 12864_2017_4382_MOESM1_ESM.docx]

## Supplementary table 1 – Databases for the BLAST sequence similarity comparisons

| Species | Entry/Release number | Database | Date |
| --- | --- | --- | --- |
| *Poecilia formosa* | Poecilia_formosa.PoeFor_5.1.22 | Ensemble | 11.11.2016 |
| *Poecilia mexicana* | GCF_001443325.1_P_mexicana-1.0 | NCBI | 24.10.2016 |
| *Poecilia latipinna* | GCF_001443285.1_P_latipinna-1.0_ | NCBI | 24.10.2016 |
| *Poecilia reticulata* | GCF_000633615.1_Guppy_female_1.0_MT | NCBI | 24.10.2016 |
| *Xiphophorus maculatus* | Xiphophorus_maculatus.Xipmac4.4.2. | Ensemble | 23.06.2015 |
| *Oryzias latipes* | Oryzias_latipes.MEDAKA1 | Ensemble | 23.06.2015 |
| *Danio rerio* | Danio_rerio.Zv9 | Ensemble | 9.06.2016 |
