# Supplementary figures and images for: The gonadal transcriptome of the unisexual Amazon molly Poecilia formosa in comparison to its sexual ancestors, Poecilia mexicana and Poecilia latipinna
Source: BMC Genomics. 2018 Jan 3;19:12. doi: 10.1186/s12864-017-4382-2 (PMC5753479; doi:10.1186/s12864-017-4382-2)

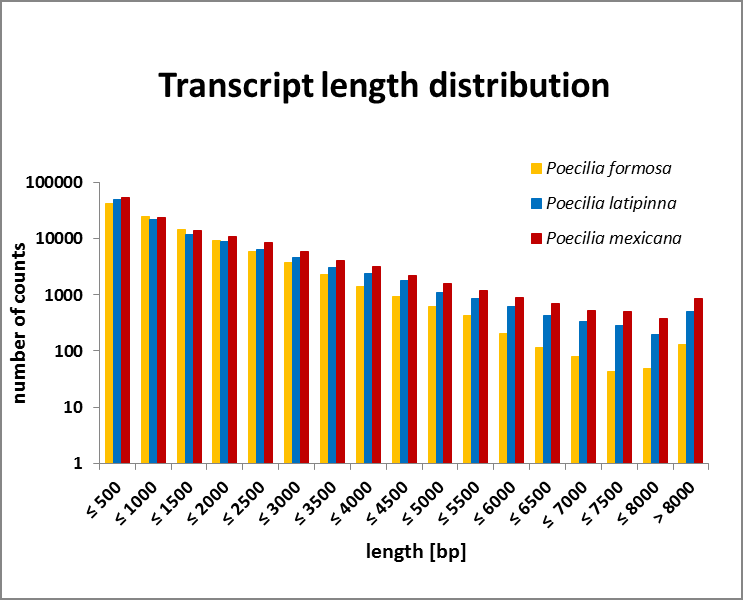

Supplement: Supplementary file 2 — Transcript length distribution for the de novo assemblies of the Amazon molly (P. formosa), the sailfin molly (P. latipinna), and the Atlantic molly (P. mexicana). (BMP 1741 kb) [file 12864_2017_4382_MOESM2_ESM.bmp]

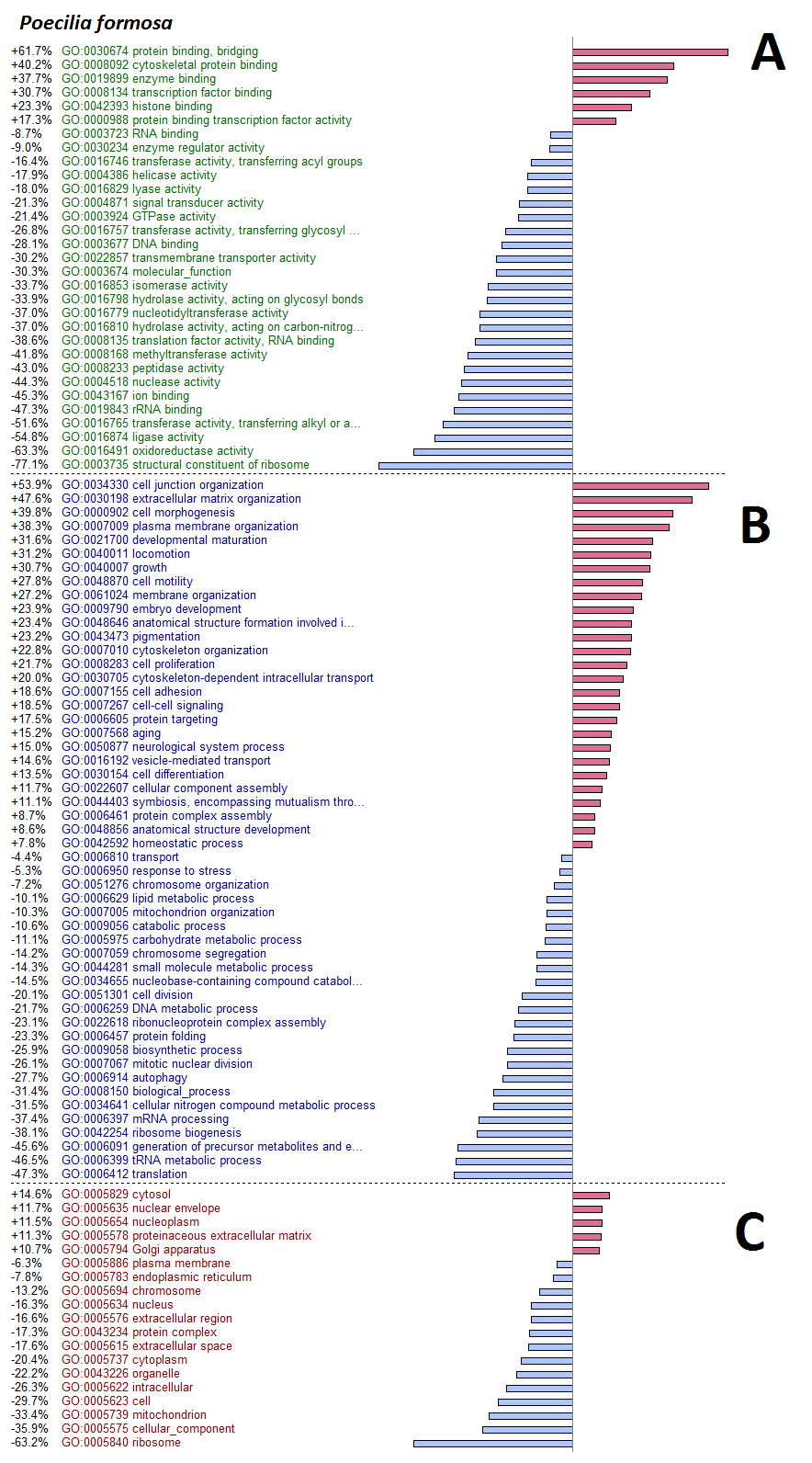

Supplement: Supplementary file 3 — Enrichment analysis of the generic GO slim terms evaluated using one-sided Fisher-Tests for P. formosa The. residues are given relative to the expected value, shown are significantly enriched (red) or depleted (blue) (p < 0,05) GOs for the three components: Molecular function (A), biological process (B), and cellular component (C). (BMP 4123 kb) [file 12864_2017_4382_MOESM3_ESM.bmp]

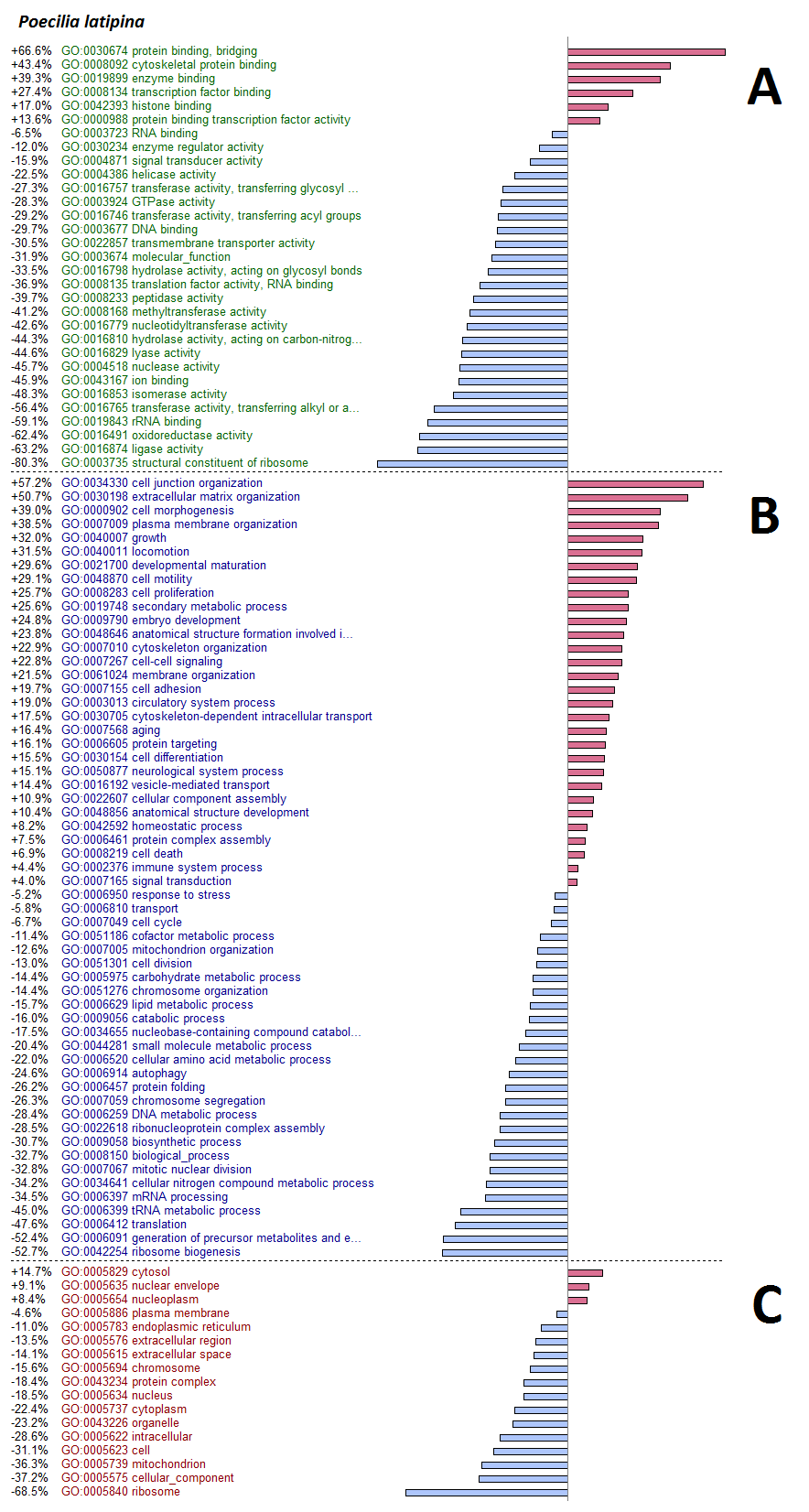

Supplement: Supplementary file 4 — Enrichment analysis of the generic GO slim terms evaluated using one-sided Fisher-Tests for P. latipinna. The residues are given relative to the expected value, shown are significantly enriched (red) or depleted (blue) (p < 0,05) GOs for the three components: Molecular function (A), biological process (B), and cellular component (C). (BMP 4278 kb) [file 12864_2017_4382_MOESM4_ESM.bmp]

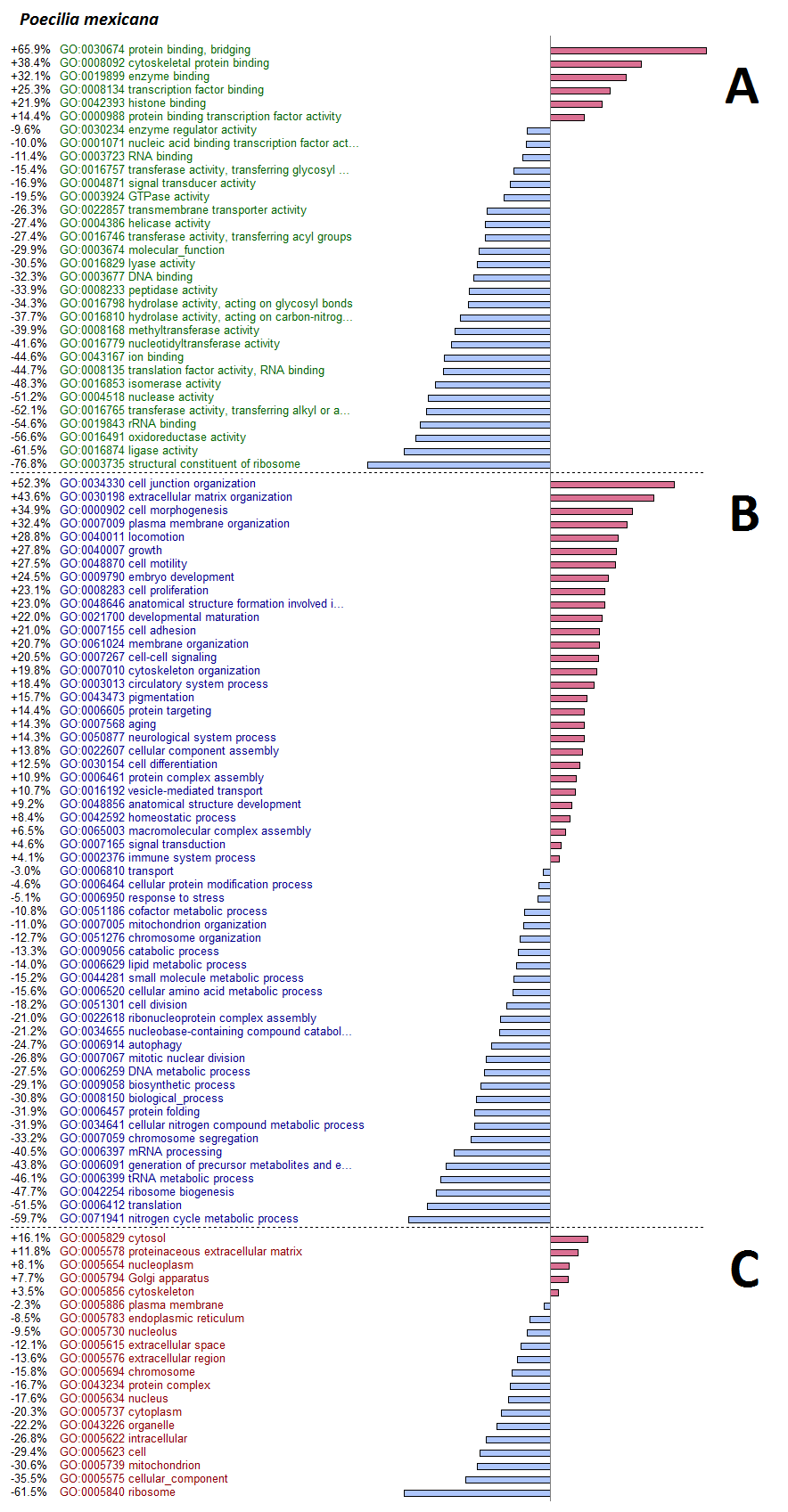

Supplement: Supplementary file 5 — Enrichment analysis of the generic GO slim terms evaluated using one-sided Fisher-Tests for P. mexicana. The residues are given relative to the expected value, shown are significantly enriched (red) or depleted (blue) (p < 0,05) GOs for the three components: Molecular function (A), biological process (B), and cellular component (C). (BMP 4395 kb) [file 12864_2017_4382_MOESM5_ESM.bmp]
