# Supplementary material for: The gonadal transcriptome of the unisexual Amazon molly Poecilia formosa in comparison to its sexual ancestors, Poecilia mexicana and Poecilia latipinna
Source: BMC Genomics. 2018 Jan 3;19:12. doi: 10.1186/s12864-017-4382-2 (PMC5753479; doi:10.1186/s12864-017-4382-2)
Supplement: Supplementary file 6 — GO terms ID, the GO term names and the corresponding genes related to reproduction and meiosis for the orthogroups only detected in the sexual species. (DOCX 17 kb) [file 12864_2017_4382_MOESM6_ESM.docx]

## Supplementary table 2 – GO terms ID, the GO term names and the corresponding genes related to reproduction and meiosis for the orthogroups only detected in the sexual species

| GO term ID | GO term name | Gene | Description |
| --- | --- | --- | --- |
| GO:0000212 | meiotic spindle organization | *clasp2* | CLIP-associating protein 2 |
| GO:0000711 | meiotic DNA repair synthesis | *sycp1* | Synaptonemal complex protein 1 |
| GO:0006311 | meiotic gene conversion | *ZNF568* | Zinc finger protein 568 |
| GO:0007136 | meiotic prophase II | *pim2* | Serine/threonine-protein kinase pim-2 |
| GO:0007143 | female meiotic division | *grap* | GRB2-related adapter protein |
| GO:0045835 | negative regulation of meiotic nuclear division | *stk32C* | Serine/threonine-protein kinase 32C |
|  |  | *dmrta2* | Doublesex- and mab-3-related transcription factor A2 |
| GO:0051307 | meiotic chromosome separation | *clasp1-a* | CLIP-associating protein 1-A |
| GO:0051447 | negative regulation of meiotic cell cycle | *stk32A* | Serine/threonine-protein kinase 32A |
|  |  | *nppc* | C-type natriuretic peptide |
| GO:0072687 | meiotic spindle | *hspa8* | Heat shock cognate 71 kDa protein |
| GO:0007538 | primary sex determination | *wt1B* | Wilms tumor protein homolog B |
|  |  | *srd5A2* | 3-oxo-5-alpha-steroid 4-dehydrogenase 2 |
| GO:0007539 | primary sex determination, soma | *onecut1* | One cut domain family member1 |
|  |  | *onecut2* | One cut domain family member 2 |
| GO:0007540 | sex determination, establishment of X:A ratio | *runx1* | Runt-related transcription factor 1 |
|  |  | *runx2* | Runt-related transcription factor 2 |
|  |  | *runx3* | Runt-related transcription factor 3 |
| GO:0007548 | sex differentiation | *clk4* | Dual specificity protein kinase CLK4 |
|  |  | *clk2* | Dual specificity protein kinase CLK2 |
|  |  | *set* | Protein SET |
|  |  | *dbx1-A* | Homeobox protein DBX1-A |
|  |  | *barhl1* | BarH-like 1 homeobox protein |
| GO:0019953 | sexual reproduction | *prad* | GTP-binding protein RAD |
|  |  | *rhoh* | Rho-related GTP-binding protein RhoH |
|  |  | *gem* | GTP-binding protein GEM |
|  |  | *rasl11b* | Ras-like protein family member 11B |
| GO:0045498 | sex comb development | *b4galt1* | Beta-1,4-galactosyltransferase 1 |
| GO:0007056 | spindle assembly involved in female meiosis | *sept2A* | Septin-2A |
|  |  | *sept4* | Septin-4 |
| GO:0007110 | meiosis I cytokinesis | *fhod1* | FH1/FH2 domain-containing protein 1 |
| GO:0016321 | female meiosis chromosome segregation | *sbk1* | Serine/threonine-protein kinase SBK1 |
| GO:0007276 | gamete generation | *cgh1* | ATP-dependent RNA helicase cgh-1 |
|  |  | *arl6* | ADP-ribosylation factor-like protein 6 |
| GO:0009566 | fertilization | *astl* | Astacin-like metalloendopeptidase |
